# Characteristic profiles of DNA epigenetic modifications in colon cancer and its predisposing conditions—benign adenomas and inflammatory bowel disease

**DOI:** 10.1186/s13148-018-0505-0

**Published:** 2018-05-30

**Authors:** Tomasz Dziaman, Daniel Gackowski, Jolanta Guz, Kinga Linowiecka, Magdalena Bodnar, Marta Starczak, Ewelina Zarakowska, Martyna Modrzejewska, Anna Szpila, Justyna Szpotan, Maciej Gawronski, Anna Labejszo, Ariel Liebert, Zbigniew Banaszkiewicz, Maria Klopocka, Marek Foksinski, Andrzej Marszalek, Ryszard Olinski

**Affiliations:** 1 Department of Clinical Biochemistry, Faculty of Pharmacy, Collegium Medicum in Bydgoszcz, Nicolaus Copernicus University in Torun, Torun, Poland; 2 Department of Clinical Pathomorphology, Faculty of Medicine, Collegium Medicum in Bydgoszcz, Nicolaus Copernicus University in Torun, Torun, Poland; 3 Department of Surgery, Faculty of Medicine, Collegium Medicum in Bydgoszcz, Nicolaus Copernicus University in Torun, Torun, Poland; 4 Department of Vascular Diseases and Internal Medicine, Faculty of Health Sciences, Collegium Medicum in Bydgoszcz, Nicolaus Copernicus University in Torun, Torun, Poland; 5 Department of Oncologic Pathology and Prophylaxis, Poznan University of Medical Sciences and Greater Poland Cancer Center, Poznan, Poland; 6 Department of Otolaryngology and Laryngeal Oncology, K. Marcinkowski University of Medical Sciences, Poznan, Poland; 7 Department of Clinical Biochemistry, Collegium Medicum in Bydgoszcz, Nicolaus Copernicus University, Karlowicza 24, 85-095 Bydgoszcz, Poland

**Keywords:** DNA epigenetic modification, Ten-eleven translocation protein, Colon cancer, Inflammatory bowel disease, Adenoma, Demethylation

## Abstract

**Background:**

Active demethylation of 5-methyl-2′-deoxycytidine (5-mdC) in DNA occurs by oxidation to 5-(hydroxymethyl)-2′-deoxycytidine (5-hmdC) and further oxidation to 5-formyl-2′-deoxycytidine (5-fdC) and 5-carboxy-2′-deoxycytidine (5-cadC), and is carried out by enzymes of the ten-eleven translocation family (TETs 1, 2, 3). Decreased level of epigenetic DNA modifications in cancer tissue may be a consequence of reduced activity/expression of TET proteins. To determine the role of epigenetic DNA modifications in colon cancer development, we analyzed their levels in normal colon and various colonic pathologies. Moreover, we determined the expressions of TETs at mRNA and protein level.

The study included material from patients with inflammatory bowel disease (IBD), benign polyps (AD), and colorectal cancer (CRC). The levels of epigenetic DNA modifications and 8-oxo-7,8-dihydro-2′-deoxyguanosine (8-oxodG) in examined tissues were determined by means of isotope-dilution automated online two-dimensional ultraperformance liquid chromatography with tandem mass spectrometry (2D-UPLC-MS/MS). The expressions of *TET* mRNA were measured with RT-qPCR, and the expressions of TET proteins were determined immunohistochemically.

**Results:**

IBD was characterized by the highest level of 8-oxodG among all analyzed tissues, as well as by a decrease in 5-hmdC and 5-mdC levels (at a midrange between normal colon and CRC). AD had the lowest levels of 5-hmdC and 5-mdC of all examined tissues and showed an increase in 8-oxodG and 5-(hydroxymethyl)-2′-deoxyuridine (5-hmdU) levels. CRC was characterized by lower levels of 5-hmdC and 5-mdC, the lowest level of 5-fdC among all analyzed tissues, and relatively high content of 5-cadC. The expression of *TET1* mRNA in CRC and AD was significantly weaker than in IBD and normal colon. Furthermore, CRC and AD showed significantly lower levels of *TET2* and *AID* mRNA than normal colonic tissue.

**Conclusions:**

Our findings suggest that a complex relationship between aberrant pattern of DNA epigenetic modification and cancer development does not depend solely on the transcriptional status of TET proteins, but also on the characteristics of premalignant/malignant cells. This study showed for the first time that the examined colonic pathologies had their unique epigenetic marks, distinguishing them from each other, as well as from normal colonic tissue. A decrease in 5-fdC level may be a characteristic feature of largely undifferentiated cancer cells.

**Electronic supplementary material:**

The online version of this article (10.1186/s13148-018-0505-0) contains supplementary material, which is available to authorized users.

## Background

During the last decade, one of the hot topics in oncogenesis was the so-called cancer epigenome, having implications for cancer promotion and progression. This, in turn, is linked with a plethora of abnormalities based on somatic heritable modifications that are not caused by alterations in primary sequence of DNA.

Methylation of cytosine, usually in CpG dinucleotides, is a key epigenetic modification exerting a profound impact on gene repression, cellular identity, and organismal fate [1]. However, equally important is an opposite reaction, DNA demethylation, resulting in activation of previously silenced genes. Although a large body of evidence suggests that active demethylation may occur in mammalian cells, its molecular background is still unclear (for review, see [2]). The most plausible mechanism behind the active demethylation of 5-methyl-2′-deoxycytidine (5-mdC) moiety in DNA involves ten-eleven translocation (TET) proteins, which catalyze oxidation of 5-mdC to form 5-(hydroxymethyl)-2′-deoxycytidine (5-hmdC) and further oxidation reactions that generate 5-formyl-2′-deoxycytidine (5-fdC) and 5-carboxy-2′-deoxycytidine (5-cadC) [2, 3]. Evidence from experimental studies supports the hypothesis that TET enzymes may be also involved in the synthesis of 5-(hydroxymethyl)-2′-deoxyuridine (5-hmdU), a molecule with epigenetic function [4].

Several recent studies showed that the level of 5-hmdC in many various types of human malignancies, including CRC, is profoundly reduced [5–7] and the degree of the reduction is proportional to tumor stage [8].

Either the mechanism or the reason behind the decrease in 5-hmdC level in cancer tissues is still not fully understood. Perhaps, this phenomenon reflects a decrease in the activity/expression of TET proteins [9]. However, it also cannot be excluded that the regulatory mechanisms of active DNA demethylation are determined by external conditions (e.g., chronic inflammation, oxidative stress, nutritional status), which results in a release of different products.

Chronic inflammation being a direct consequence of inflammatory bowel disease (IBD) is considered the most important etiological factor of sporadic colorectal malignancies [10]. Epigenetic modification of DNA is a dynamic molecular process, being a form of response to inflammation-related environmental/metabolic changes.

The downstream steps of active demethylation process may be, at least partially, responsible for the loss of 5-mdC. Furthermore, 5-fdC and 5-cadC were shown to be recognized by a larger number of proteins than 5-hmdC, despite markedly higher level of the latter [11, 12].

In the vast majority of previous studies, 5-hmdC, 5-fdC, and 5-cadC were determined semi-quantitatively, by means of immunohistochemistry; consequently, the results of these might be biased due to ultra-low content of these modifications in genomic DNA of the tumor [7]. It should be also stressed that the accuracy of immunohistochemical studies depends largely on the sensitivity/specificity of antibodies against a given modification.

In our present study, instead of using a semiquantitive method with anti-5-hmdC antibodies, we determined 5-mdC, 5-hmdC, 5-fdC, 5-cadC, and 5-hmU with a highly specific and highly sensitive method developed recently in our laboratory: isotope-dilution automated online two-dimensional ultra-performance liquid chromatography with tandem mass spectrometry (2D-UPLC-MS/MS) [13].

To provide a better insight in the relationship between epigenetic DNA modifications and factors which may influence formation thereof and to determine their role in CRC development, we analyzed their levels in normal colon and various colonic pathologies, which predispose to CRC development. Moreover, we determined the expressions of TETs and AID at mRNA and protein level.

The study included samples from patients with CRC (*n* = 97, both from the tumor and from normal colonic tissue), colon adenomas (AD, *n* = 39), and IBD (*n* = 49). Since both CRC and chronic inflammation are associated with oxidative stress, aside from the epigenetic DNA modifications, we also determined an established marker of oxidatively modified DNA, 8-oxodG. The rationale of the study was to fill the gap in existing knowledge, explaining how conditions which predispose to CRC development can influence the synthesis of TET-mediated DNA modifications and oxidatively modified DNA.

## Methods

### Study group

The study material originated from three groups of patients with (1) IBD (*n* = 49, median age 35 years, 53% of women), (2) AD, i.e., histologically confirmed adenoma tubulare (90%) or adenoma tubulovillosum (10%) (*n* = 39, median age 65 years, 46% of women), and (3) CRC, i.e., histologically confirmed stage A (8%), stage B (45%), stage C (29%), or stage D (9%) adenocarcinoma, or malignant polyps (9%) (*n* = 97, median age 65 years, 46% of women). None of the study subjects were related with one another, and all of them were Caucasians. All participants of the study were recruited in a hospital setting (Collegium Medicum, Nicolaus Copernicus University, Bydgoszcz, Poland) and subjected to colonoscopy. At the enrollment, all subjects completed a questionnaire containing information about their demographics, smoking, diet, and medical history. The study groups were matched for eating habits, age, body weight, and smoking status. No significant intergroup differences were found in terms of body weight and body stature of male and female subjects. To make the study groups even more homogenous, the subjects who reported overeating or use of dietary supplements during a month preceding the study were not included in the analysis. The questionnaire survey was conducted by the team physician (Dr. Banaszkiewicz, Dr. Klopocka).

### Preparation of tissue microarrays (TMA) for immunochemical analysis

Immunohistochemical studies were performed using archived formaldehyde-fixed paraffin-embedded (FFPE) tissue sections derived in the Department of Clinical Pathomorphology, Collegium Medicum in Bydgoszcz, Nicolaus Copernicus University in Torun.

Hematoxylin and eosin (H&E)-stained microscopic slides of archived FFPE tissue sections (donor blocks) were used to identify representative tumor areas with at least 80% tumor cells. Then, two such regions, each 2 mm in diameter, were transferred from the donor blocks to a recipient TMA block using an automated tissue arrayer (TMA Master3D HISTECH, Budapest, Hungary). The same procedure was repeated for normal tissue located at least 2 cm from the tumor resection margin. Then, another set of H&E-stained slides was prepared to verify the accuracy of the TMA blocks. TMA blocks were verified and double-checked by two independent pathologists.

### Immunohistochemistry

Immunohistochemical staining was carried out as described elsewhere [14–16], and the results were standardized against a series of positive and negative controls. Positive control staining was performed on a model tissue section selected according to The Human Protein Atlas (http://www.proteinatlas.org) [17] and antibody specification (Additional file 1: Table S1) and the antibody datasheet. Negative controls were prepared from the examined tissues treated with 1% solution of bovine serum albumin (BSA) in phosphate buffered saline (PBS), instead of the primary antibody. Paraffin TMA blocks and archived FFPE tissue sections were cut with a manual rotary microtome (AccuCut, Sakura, Torrance, USA) to obtain 4-μm slices, which were then processed routinely and mounted on extra adhesive slides (SuperFrostPlus, MenzelGlasser, Braunschweig, Germany).

The deparaffinization, rehydration, and antigen retrieval were carried out in PT-Link system (Dako, Agilent Technologies, USA). The slides were heated for 20 min in Epitope Retrieval Solution high-pH (95–98 °C; Dako, Agilent Technologies). Then, the activity of endogenous peroxidase was blocked by a 15-min incubation with 3% H_2_O_2_ solution, and non-specific binding was eliminated by a 15-min incubation with 5% BSA solution; both reactions were carried out at room temperature. Subsequently, the slides were incubated with primary antibodies against TET1, TET2, and TET3 (specified in Additional file 1: Table S1). The antibody complexes were detected with EnVision Flex Anti-Mouse/Rabbit HRP-Labeled Polymer (Dako, Agilent Technologies) and localized using 3–3′diaminobenzidine (DAB) as a chromogen. Finally, the slides were counterstained with hematoxylin, subsequently dehydrated, cleared in series of xylenes, and coverslipped using mounting medium (Dako, Agilent Technologies).

### Evaluation of protein expression based on immunohistochemical staining

Each slide was examined under ECLIPSE E400 light microscope (Nikon Instruments Europe, Amsterdam, Netherlands) with the low-power (20×) objective. The result of immunohistochemical staining was expressed according to Immunoreactive Remmele-Stegner (IRS) score [18], described in detail in our previous papers [14–16, 19]. Total IRC score (from 0 to 12) was obtained by multiplying the staining intensity score (0—negative, 1—weak staining, 2—moderate staining, 3—strong staining) by the relative proportion of immunolabeled specimen area (0—none; 1—less than 10%; 2—10 to 50%; 3—50 to 80%; 4—at least 80%).

### Extraction of DNA from tissues and its hydrolysis to deoxynucleosides

DNA from examined fresh frozen tissues was isolated as described elsewhere [20], with some modifications. Isolated DNA was dissolved in 100 mM ammonium acetate (Sigma-Aldrich) containing 0.1 mM ZnCl_2_ (pH 4.3). The dissolved DNA samples (50 μl) were mixed with 1 U of nuclease P1 (Sigma-Aldrich) and tetrahydrouridine (Calbiochem) (as cytidine deaminase inhibitor, 10 μg per sample) and incubated at 37 °C for 1 h. Subsequently, 12 μl 5% (*v*/*v*) NH_4_OH (JT Baker) and 1.3 U of alkaline phosphatase (Sigma-Aldrich) were added to each sample following 1-h incubation at 37 °C. Finally, all DNA hydrolysates were acidified with CH_3_COOH (Sigma-Aldrich) (to final *v*/*v* concentration of 2%) and ultrafiltered prior to injection.

### Isolation of DNA and determination of epigenetic modifications and 8-oxodG in DNA isolates

The methodology used to determine 5-methyl-2′-deoxycytidine (5-mdC), 5-hydroxymethyl-2′-deoxycytidine (5-hmdC), 5-formyl-2′-deoxycytidine (5-fdC), 5-carboxy-2’deoxycytidine (5-cadC), 5-(hydroxymethyl)-2′-deoxyuridine (5-hmdU), and 8-oxodG levels by means of 2D-UPLC-MS/MS has been described elsewhere [13]. Transition patterns and specific detector settings for all analyzed compounds are presented in the Additional file 2: Table S2.

### Gene expression analysis

Isolated leukocytes were stored at − 80 °C until the analysis. RNA was isolated with MagNA Pure 2.0 (Roche) following the standard procedures. Concentration and purity of RNA aliquots were verified spectrophotometrically with NanoDrop 2000 (Thermo Scientific). A_260_/A_280_ ratio was used as an indicator of protein contamination and A_260_/A_230_ ratio as a measure of contamination with polysaccharides, phenol, and/or chaotropic salts. Quality and integrity of total RNA were assessed by visualization of 28S/18S/5.8*S rRNA* band pattern in a 1.2% agarose gel. Non-denaturing electrophoresis was carried out at 95 V for 20 min in TBE buffer (Tris – Boric Acid – EDTA). The gel was stained with ethidium bromide or SimplySafe and visualized using GBox EF Gel Documentation System (SynGene). Purified RNA was stored at − 80 °C. The samples with RNA concentrations greater than 50 ng/μl were qualified for further analysis. 0.5 microgram of total RNA from each sample (in 20-μl volume) was used for cDNA synthesis by reverse transcription with High-Capacity cDNA Reverse Transcription Kit (Applied Biosystems, catalog no. 43-688-14), according to the manufacturer’s instruction. The reaction was carried out with Mastercycler Nexus Gradient thermocycler (Eppendorf). To exclude contamination with genomic DNA, reverse transcriptase reaction included also a negative control. cDNA was either used for qPCR setup immediately after obtaining or stored at − 20 °C. The RT-qPCR complies with the Minimum Information for Publication of Quantitative Real-time PCR Experiments (MIQE) guidelines. Three gene transcripts, *TET1*, *TET2*, and *TET3*, were analyzed by relative quantitative RT-PCR (RT-qPCR) with relevant primers and short hydrolysis probes substituted with Locked Nucleic Acids from the Universal Probe Library (UPL, Roche) (see: Additional file 3: Table S3). The probes were labeled with fluorescein (FAM) at the 5′-end and with a dark quencher dye at the 3′-end. Expressions of target genes were normalized for two selected reference genes, *HMBS* (GeneID: 3145) and *TBP* (GeneID: 6908), using UPL Ready Assay #100092149 and #100092158, respectively. Real-time PCR mixes (in 20 μl volumes) were prepared from cDNA following the standard procedures for LightCycler480 Probes Master (Roche), provided with the reagent set. The reactions were carried out on 96-well plates. Aside from the proper samples, each plate included also no-template control and no-RT control. Quantitative real-time PCR was carried out with LightCycler 480 II, using the following cycling parameters: 10 s at 95 °C, followed by 45 repeats 10 s each at 95 °C; 30 s at 58 °C; and finally, 1 s at 72 °C with acquisition mode (parameters of wavelength excitation and detection equal 465 and 510 nm, respectively). The reaction for each gene was standardized against a standard curve, to estimate amplification efficiency. Standardization procedure included preparation of 10-fold serial dilutions with controlled relative amount of targeted template. The efficiency of amplification was assessed based on a slope of the standard curve. Standard dilutions were amplified in separate wells, but within the same run. Then, the samples were subjected to qPCR with measurement of *C*_t_, and amplification efficiencies were automatically calculated and displayed on the analysis window of LightCycler 480 software, version 1.5.1.62 (Roche). The same software was also used for sample setup, real-time PCR analysis, and calculation of relative *C*_t_ values referred to as “ratios.”

### Statistical methods

The results are presented as medians, interquartile ranges, and non-outlier ranges. Normal distribution of the study variables was verified with Kolmogorov-Smirnov test with Lilliefors correction and based on visual inspection of plotted histograms. Variables with non-normal distributions (5-hmdC, 5-fdC, 5-cadC, 5-hmdU, 8-oxodG concentration and *TET*s, *AID* mRNA expression) were subjected to Box-Cox transformation prior to statistical analyses with parametric tests. Normalized data were subjected to one-way analysis of variance (ANOVA) followed by LSD and Tukey post hoc tests. Associations between pairs of variables were assessed based on Pearson correlation coefficients for raw or normalized data, where applicable. All statistical transformations and analyses were carried out with STATISTICA 13.1 PL [Dell Inc. (2016). Dell Statistica (data analysis software system), version 13. software.dell.com.]. The results were considered statistically significant at *P* values lower than 0.05.

## Results

### Levels of epigenetic modifications and 8-oxodGuo in DNA from tissue specimens

**Fig. 1:**
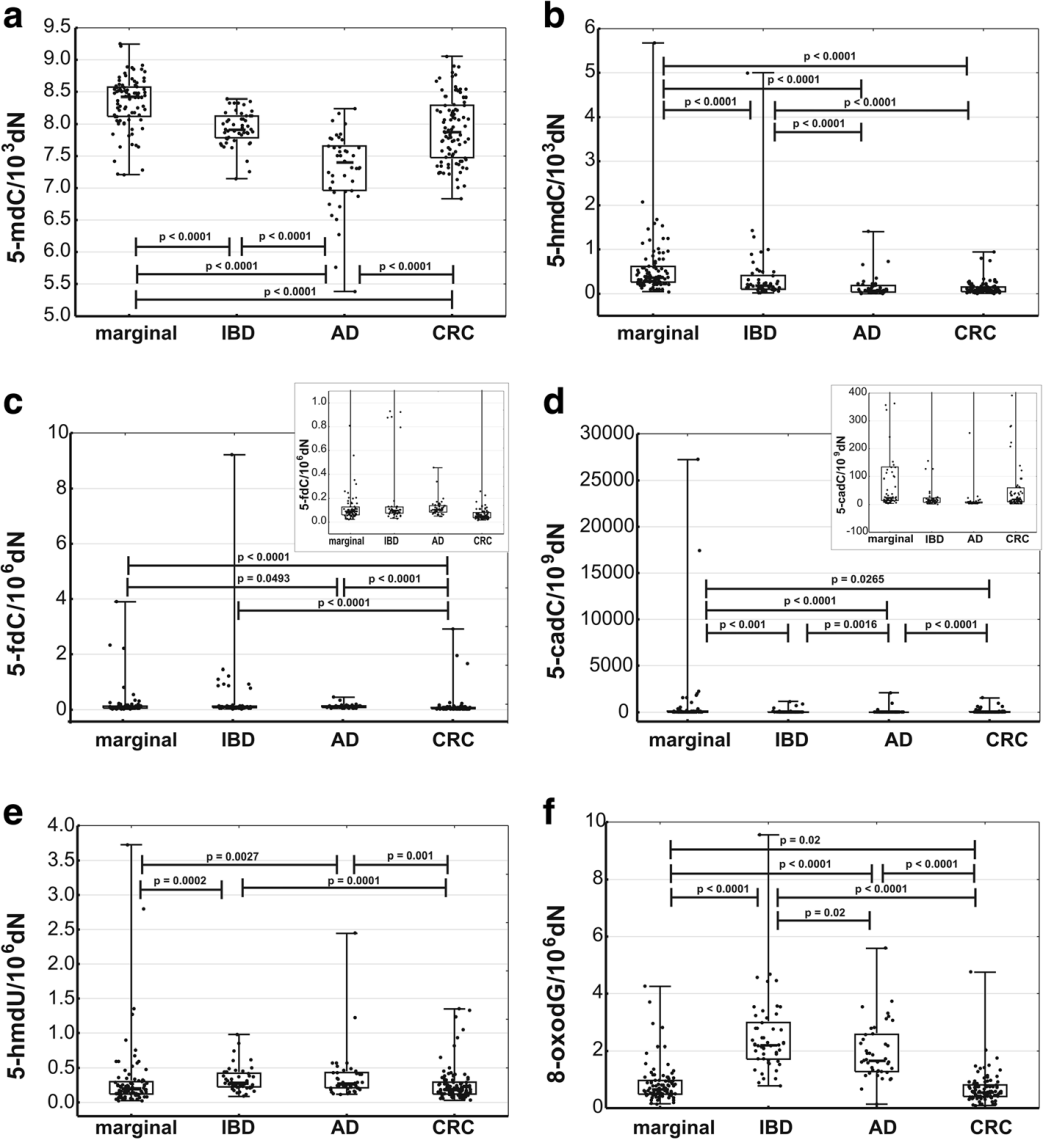
Levels of DNA epigenetic modifications—5-mdC (**a**), 8-oxodG (**b**), 5-hmdC (**c**), 5-fdC (**d**), 5-cadC (**e**), and 5-hmdU (**f**) in normal colonic tissue (*n* = 90); inflammatory lesions, IBD (*n* = 49); polyps, AD (*n* = 39); and cancer tissue, CRC (*n* = 97). Marker in the center of the box represents median value. The length of each box (IQR, interquartile range) represents the range of values for 50% of the most typical observations, and its edges correspond to the first and third quartile. Whiskers represent variance outside the upper and lower quartile. *P* value was determined with Mann-Whitney *U* test

The highest levels of 5-mdC and 5-hmdC were found in normal colonic tissue, followed by IBD, AD, and CRC specimens (Fig. 1a, b); the level of 5-mdC in AD turned out to be significantly lower than in other tissues. In turn, CRC specimens were characterized by significantly lower levels of 5-fdC than other samples (Fig. 1c). The level of 5-cadC in AD was significantly (2- to 2.5-fold) lower than in other tissues; in turn, the highest level of this modification was found in normal colonic tissue (Fig. 1d). We also analyzed possible association between level of the epigenetic modifications and tumor progression reflected in tumor stage from A to D. Significant decrease of 5-mdC, 5-hmdC, and 5-fdC was characteristic for early stage of CRC development (stage A), and no further changes were observed along the disease progression.

The highest levels of 5-hmdU and 8-oxodG were observed in IBD and AD and the lowest in CRC and normal colonic tissue; also, these intergroup differences were statistically significant (Fig. 1e, f).

**Fig. 2:**
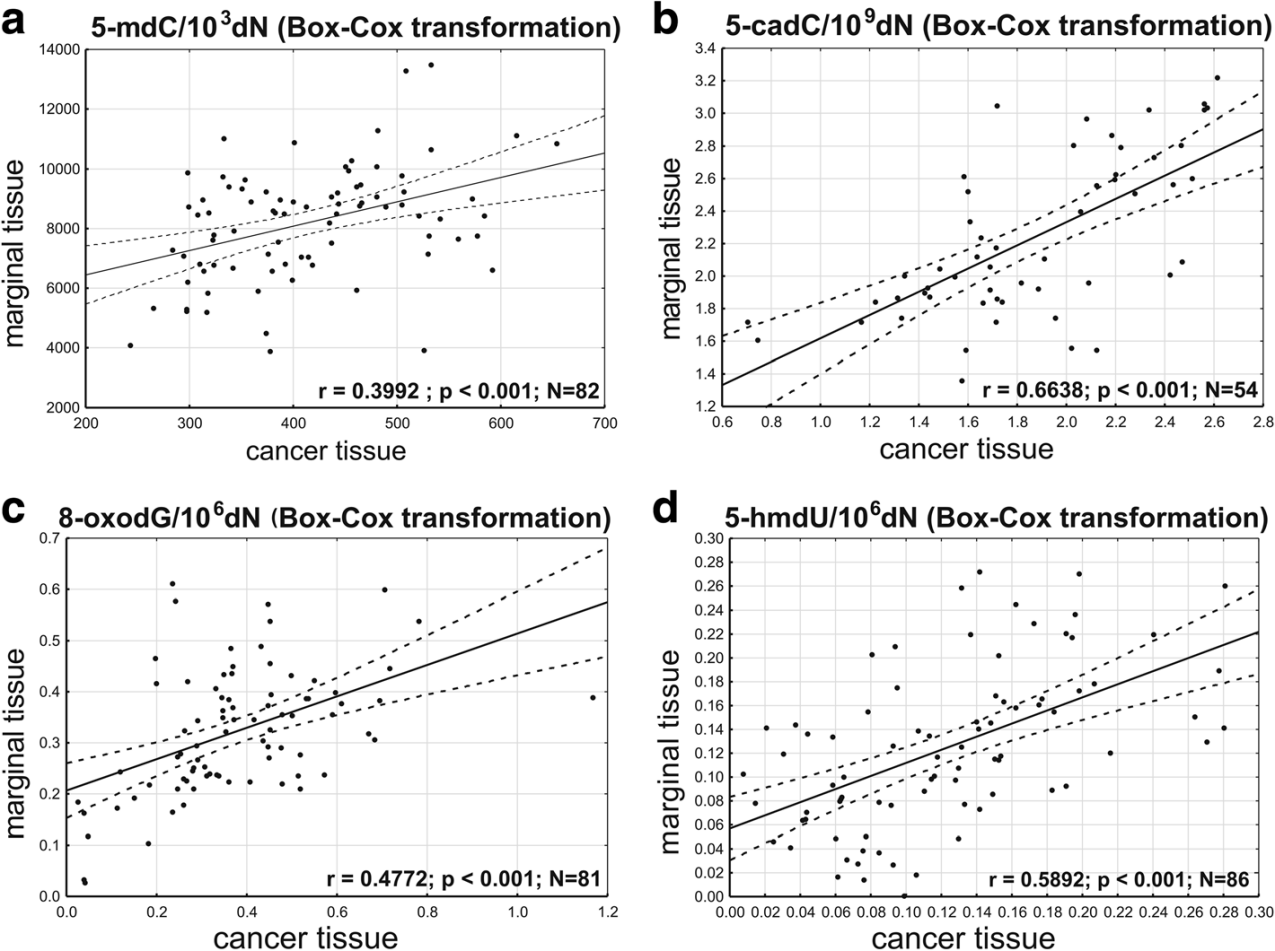
Correlations between the levels of DNA epigenetic modifications, 5-mdC (**a**), 5-cadC (**b**), 8-oxodG (**c**), and 5-hmdU (**d**), in normal colonic tissue and cancer tissue

Furthermore, significant correlations were found in the levels of 5-mdC, 5-cadC, 8-oxodG, and 5-hmdU between CRC and normal colon (Fig. 2).

We have analyzed relationship/correlation between age and 5-hmCyt (and other modifications). However in CRC patients as well as in other groups, no such correlation was found.

### Expression of TET and AID mRNA

**Fig. 3:**
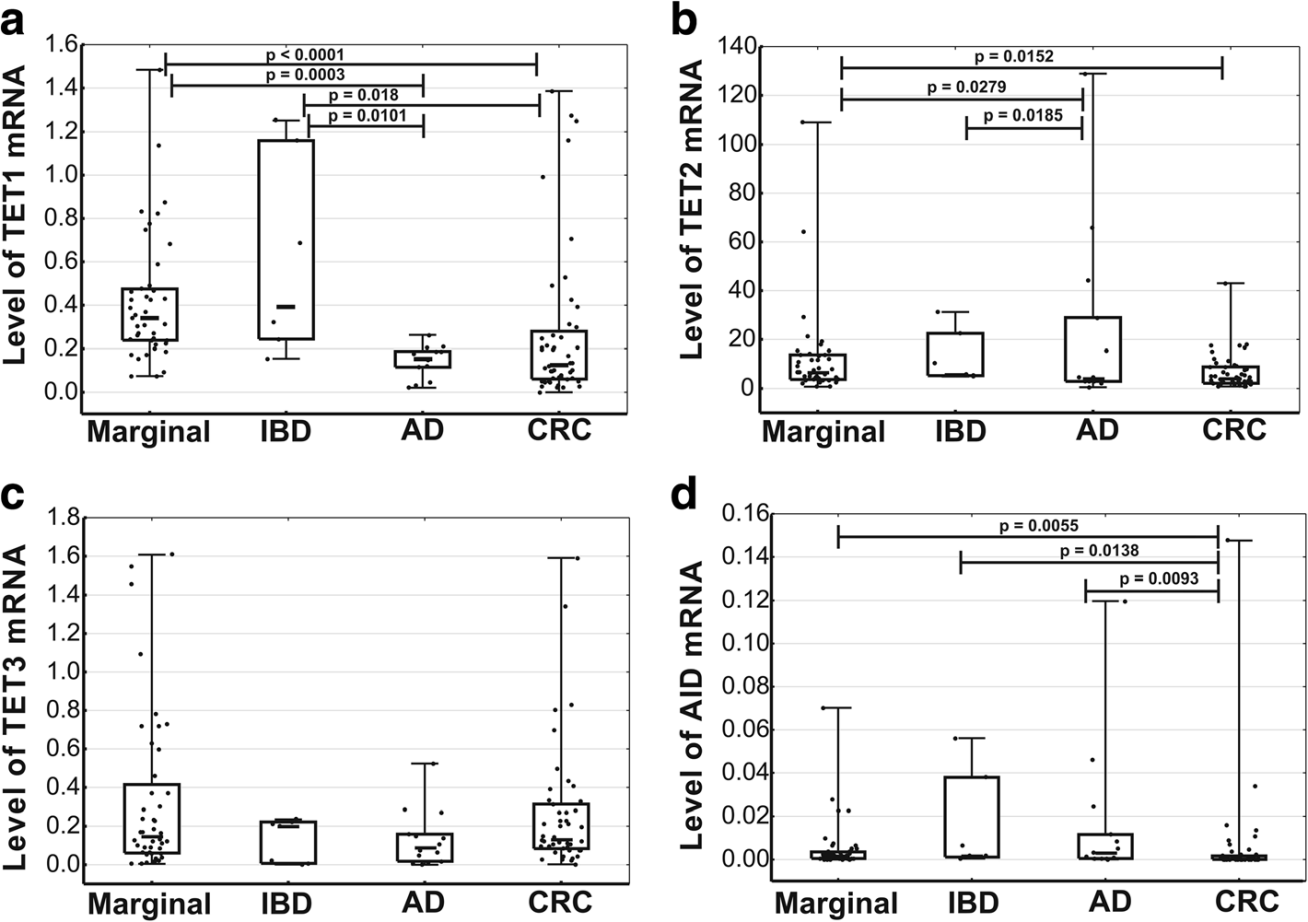
Expressions of *TET1* (**a**), *TET2* (**b**), *TET3* (**c**), and *AID* (**d**) mRNA in normal colonic tissue, cancer tissue (CRC, *n* = 49), inflammatory lesions (IBD, *n* = 7), and polyps (AD, *n* = 14). Marker in the center of the box represents median value. The length of each box (IQR, interquartile range) represents the range of values for 50% of the most typical observations, and its edges correspond to the first and third quartile. Whiskers represent variance outside the upper and lower quartile. *P* value was determined with Mann-Whitney *U* test

The expression of *TET1* in AD and CRC was significantly weaker than in normal colonic tissue and IBD (Fig. 3a). The expression of *TET2* in normal colonic tissue was significantly weaker than in AD and CRC; moreover, a significant difference was found in *TET2* expressions in IBD and AD (Fig. 3b). The examined tissues did not differ significantly in terms of *TET3* expressions (Fig. 3c). Irrespective of the examined tissue, the levels of *AID* mRNA were very low or below the detection threshold. Nevertheless, the levels of *AID* mRNA in CRC turned out to be significantly lower than in other tissues (Fig. 3d). No statistically significant correlations were found between TETs expression and epigenetic DNA modifications (data not shown).

### Immunohistochemical analysis of protein expression

**Fig. 4:**
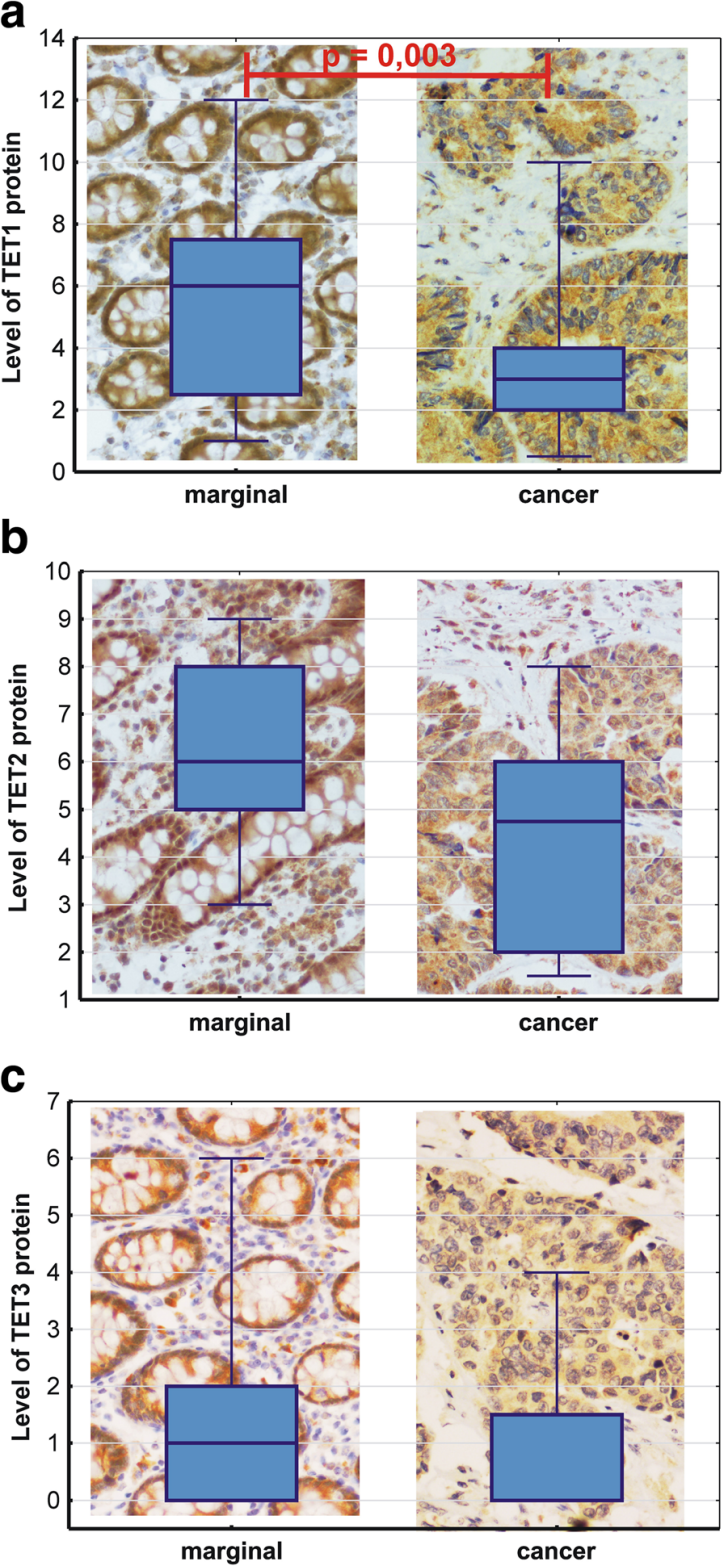
Expressions of TET1 (**a**), TET2 (**b**), and TET3 (**c**) protein in normal colonic tissue and cancer tissue (CRC, *n* = 19). Marker in the center of the box represents median value. The length of each box (IQR, interquartile range) represents the range of values for 50% of the most typical observations, and its edges correspond to the first and third quartile. Whiskers represent variance outside the upper and lower quartile. *P* value was determined with Mann-Whitney *U* test

Immunoreactivity to anti-TET1 antibodies in CRC turned out to be significantly lower than in normal colonic tissue (Fig. 4). In the case of TET2, immunohistochemical analysis was on the borderline of statistical significance with *p* = 0.06.

## Discussion

Although a molecular link between adenomas, chronic inflammation, and carcinogenesis is still not completely understood, a major contributor to CRC development seems to be aberrant methylation of DNA and oxidatively damaged DNA. A growing body of evidence suggests that decreased levels of 5-mdC (for review, see: [21]) and 5-hmdC [22] may be found not only in human malignancies but also in their precursor lesions, such as adenomas. This implies that the level of this modification may decrease gradually throughout carcinogenesis. However, it is still unclear if hypomethylation is a late or early event in cancer development, and whether this process is directly involved in carcinogenesis (for review, see: [21]). Therefore, the aim of this study was to verify if CRC and its precursor lesions differ from normal colonic tissue in the levels of DNA epigenetic modifications.

Similar to previous studies, we demonstrated that 5-hmdC level in CRC was several times lower than in normal colonic tissue. However, the level of this modification in cancer precursor lesions still raises some controversies. In some studies, the levels of 5-hmdC in benign lesions were shown to be lower than in normal tissues [7, 22]. However, in a recent study, the results of which were published in *Cell* [6], melanocytes forming benign nevi showed relatively high levels of 5-hmdC, whereas a significant decrease or complete loss of this epigenetic mark was observed in melanoma cells. It should be stressed that in all these studies, 5-hmdC was determined with a less accurate semiquantitative method. Our present study, involving highly accurate quantitative technique for 5-hmdC determination, demonstrated that the level of this modification in AD and CRC was essentially the same, approximately four times lower than in normal colonic tissue. These findings are consistent with the results published by Uribe-Lewis et al. [22], who also showed that 5-hmdC levels in CRC and adenoma were substantially lower than in normal colon. However, to the best of our knowledge, our present study was the first one to show that the level of 5-hmdC in IBD was significantly lower than in normal colonic tissue, at a midrange between the values found in this material and in CRC (Fig. 1b).

Interestingly, a significant decrease in 5-fdC content was observed solely in CRC, and the level of this modification in both types of precursor lesions, IBD and AD, was essentially the same as in normal colonic tissue (Fig. 1c). Furthermore, both IBD and AD were characterized by significantly higher levels of 8-oxodG (the marker of oxidative stress) than CRC and normal colon (Fig. 1f). It should be remembered that the induction of oxidative stress in a cell culture was previously shown to contribute to a decrease in 5-hmdC level [23].

The level of another higher-order oxidative modification of 5-mdC, i.e., 5-cadC, was significantly higher in CRC than in AD or IBD (Fig. 1a, d). Furthermore, we found a significant correlation between the levels of this modification in CRC and normal colonic tissue (Fig. 2). Previous semiquantitative studies with specific antibodies demonstrated elevated levels of 5-cadC in human breast cancer and gliomas [24].

Recent evidence suggests that oncogenic transcription factors, Myc and Max, and perhaps also an array of regulatory proteins, can specifically recognize 5-cadC, having lesser affinity for 5-fdC and showing only a trace of affinity towards 5-mdC and 5-hmdC. It should be remembered that dysregulation of MYC-MAX transcriptional network is a common mechanism driving progression of human malignancies [25]. This may at least partially explain higher levels of 5-cadC found in cancer tissue. Moreover, Xiong et al. [26] showed recently that Sall4, an oncogenic protein being overexpressed in colon cancer [27], may cooperate with TET2, catalyzing oxidation of 5-hmdC and contributing to formation of 5-cadC. Another study demonstrated that TET3 may specifically bind to 5-cadC, initiating BER pathway and thus activating the process of demethylation [28].

**Fig. 5:**
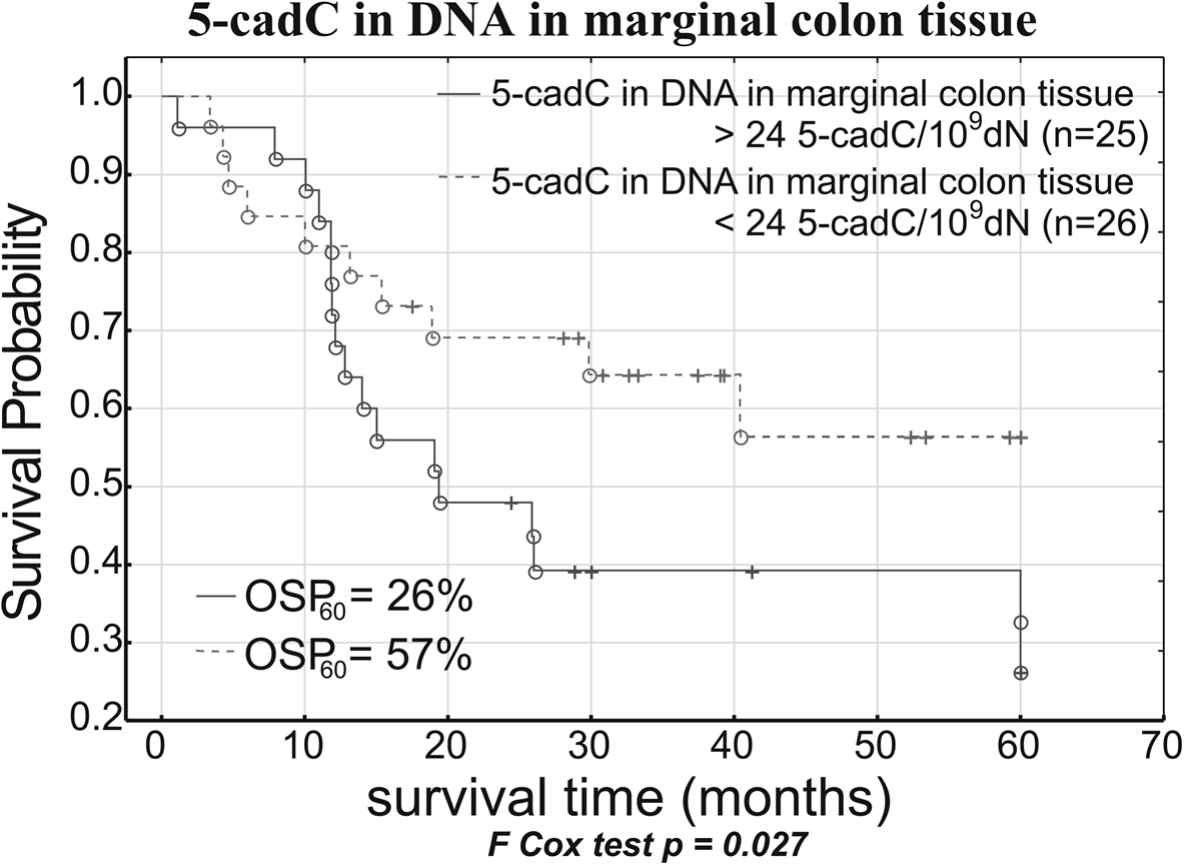
Relation between the level of 5-cadC in marginal colon tissue and survival of CRC patients following surgery

Intriguingly, the analyses of associations between overall survival and the levels of epigenetic modifications in CRC patients demonstrated that the only correlation of longer survival was low level of 5-cadC in marginal tissue (Fig. 5). Hence, an important question arises why this parameter is a meaningful predictor of longer survival in cancer patients? Although histopathological examination did not demonstrate presence of cancer cells in marginal/normal tissue, the molecular assays detected the cells being clonally related to the tumor (field cancerization) [29]. Moreover, in the case of CRC colon marginal tissue, field cancerization may involve up to 10-cm patches [30]. Therefore, the relatively large specimen of marginal tissue used for DNA isolation likely corresponded to the area of field cancerization. Considering a strong correlation between 5-cadC levels in CRC and normal colonic tissue and a relatively high content of this modification in CRC, one can expect that marginal tissue with lower level of 5-cadC is less likely to contain cancer cells.

Both CRC and its precursor lesions, especially AD, showed significantly lower levels of 5-mdC than normal colonic tissue (Fig. 1). A dramatic decrease in 5-mdC and 5-hmdC levels in AD may contribute to genomic instability and thus represent a decisive step in CRC development. Interestingly, a substantial decrease in the levels of these modifications in AD (observed also in IBD specimens) co-existed with an increase in 5-hmU, 8-oxodG, and 5-fdC content. This suggests that the decrease in 5-mdC level observed in cancer precursor lesions may be associated with the recently proposed phenomenon of processive DNA demethylation. Plausibly, 5-fdC, 5-hmd, and perhaps also 8-oxodG initiate processive demethylation of DNA, as proposed by Franchini et al. [31, 32]. In line with this hypothesis, an alternative pathway, the so-called processive DNA demethylation, exists aside from the active process involved in local and specific demethylation of DNA. According to the authors of this hypothesis, a single initiating event (such as a certain mismatch, e.g., 5-hmUra-G) may trigger processive demethylation of numerous 5-mdCs (and perhaps also 5-hmdCs) on the same locus via long-path BER, DNA mismatch repair (MMR), or nucleotide excision repair (NER) pathway. Recent experiments with cell-free extracts and circular heteroduplex DNA substrate demonstrated that 5-hmU may trigger the removal of distant epigenetic modifications (5-mdC and 5-hmdC) on MMR- and long-path BER-dependent pathway [33].

Our present study showed that the expression of *TET1* mRNA in CRC and AD was significantly weaker than in IBD and normal colon (Fig. 3). Furthermore, CRC and AD showed significantly lower levels of *TET2* and *AID* mRNA than normal colonic tissue. However, at a protein level, the only significant difference between the examined tissues was found in the case of *TET1*, significantly more abundant in normal colon than in CRC.

A main factor contributing to a decrease in the activity of TET proteins are mutations in catalytic domains of these enzymes [34]. Another reason behind the reduced activity of TETs may be an inhibitory effect of accumulated onco-metabolites, such as 2-OH-glutarate [35, 36], resulting primarily from the presence of IDH1/2 mutations. However, these mutations were observed mainly in hematopoietic malignances and are rare or completely absent in solid tumors, such as CRC [37–39]. This implies that a decrease in 5-hmdC level may be caused by other factors than TET/IDH mutations, for example oxidative stress. Indeed, recent evidence suggests that oxidative stress may contribute to post-translational modulation of TET2 [40]. Recently, it was demonstrated that the inhibition of TET proteins may be a direct consequence of hypoxia [41]. Hypoxia is a common phenomenon in solid tumors, which may at least partially explain a decrease in 5-hmdC and 5-fdC levels observed in CRC. Interestingly, hypoxia increases overall oxidative stress and can change redox status of the cell [42].

Since the shape of TET co-substrates (2-ketoglutarate, Fe^+2^) depends on the redox state of the cell, the change in the activity of these enzymes may reflect the severity of oxidative stress. Furthermore, it cannot be excluded that also superoxide (O^−2^), an anion radical of dioxygen and the precursor of free radicals, plays an important role in TET-mediated active DNA demethylation [43, 44]. Thus, changes in the activity of TET proteins may result from a persistent increase in the severity of oxidative stress, which promotes aberrant generation of DNA epigenetic modifications during iterative oxidation of 5-mdC. In this study, we found elevated levels of 8-oxodG in IBD and AD. Of note, level of 8-oxodG in DNA may directly inform about oxidative stress in nuclei of cells, where epigenetic processes take place. Recently, it was also demonstrated that 8-oxodG may serve as a demethylation signal [45]: binding to 8-oxodG; OGG1 glycosylase may recruit TET1 which in turn may be involved in specific DNA demethylation in response to oxidative stress/oxidatively damaged DNA. This may at least partially explain a decrease in 5-mdC level observed in AD and IBD, i.e., in precursor lesions characterized by elevated levels of 8-oxodG.

## Conclusion

Our hereby presented findings suggest that a complex relationship between aberrant pattern of DNA epigenetic modification and cancer development does not depend solely on the transcriptional status of TET proteins, but also on the characteristics of premalignant/malignant cells. This in turn implies that epigenetic modification of DNA is linked to oxidative stress. However, the exact character of this complex relationship is still poorly understood.

Our findings are consistent with the results of previous studies, showing that aberrant methylation of DNA occurs at very early stages of CRC development. Moreover, the hereby presented data add to existing evidence, showing that a decrease in the level of epigenetic marks is characteristic for early stages of CRC development, and further progression of the tumor is not associated with any additional changes in these parameters. To the best of our knowledge, this study was the first one to show that CRC, AD, and IBD had their unique epigenetic marks, distinguishing them from each other as well as from normal colonic tissue: (i) IBD was characterized by the highest level of 8-oxodG among all analyzed tissues, as well as by a decrease in 5-hmdC and 5-mdC levels (at a midrange between normal colon and CRC); (ii) AD had the lowest levels of 5-hmdC and 5-mdC of all examined tissues and showed an increase in 8-oxodG and 5-hmdU levels; (iii) CRC was characterized by lower levels of 5-hmdC and 5-mdC, the lowest level of 5-fdC among all analyzed tissues, and relatively high content of 5-cadC. This implies that a decrease in 5-hmdC level is not a unique feature of CRC (as previously reported) and can be also found in its precursor lesions, in particular in AD. The mechanism behind a substantial decrease in 5-fdC generation at advanced stages of carcinogenesis is still unclear, and the same refers to the consequences of this phenomenon. A recent observation that 5-fdC is rich in active enhancers involved in tissue development/differentiation [46] sheds a new light on these relationships, implying that reduced level of this modification may be a characteristic feature of largely undifferentiated cancer cells.

It cannot be excluded that the analysis of a larger spectrum of DNA epigenetic modifications, rather than solely 5-hmdC, supported by a transcriptional information, might provide a better insight in carcinogenesis, risk factors of CRC, and perhaps also therapy of this malignancy.

## Additional files

Additional file 1: **Table S1.** Characteristics of antibodies used in immunohistochemical staining. (PDF 499 kb)

Additional file 2: **Table S2.** Transition patterns specific detector settings and sources of standards for analyzed compounds (relative response ratio = area under the peak of qualifierion/area under the peak of quantifier ion). (PDF 82 kb)

Additional file 3: **Table S3.** Primers and short hydrolysis probes used for TETs and AID mRNA expression analysis. (PDF 442 kb)

## Abbreviations

2D-UPLC-MS/MS: Two-dimensional ultra-performance liquid chromatography with tandem mass spectrometry
5-cadC: 5-Carboxy-2’deoxycytidine
5-fdC: 5-Formyl-2′-deoxycytidine
5-hmdC: 5-Hydroxymethyl-2′-deoxycytidine
5-hmdU: 5-(Hydroxymethyl)-2′-deoxyuridine
5-mdC: 5-Methyl-2′-deoxycytidine
8-oxodG: 8-Oxo-2′-deoxyguanosine
AD: Adenoma
AID: Activation-induced cytidine deaminase protein
CRC: Colorectal cancer
FFPE: Formaldehyde-fixed paraffin-embedded
IBD: Inflammatory bowel disease
TET: Ten-eleven translocation protein
TMA: Tissue microarrays
