# Supplementary material for: Characteristic profiles of DNA epigenetic modifications in colon cancer and its predisposing conditions—benign adenomas and inflammatory bowel disease
Source: Clin Epigenetics. 2018 May 30;10:72. doi: 10.1186/s13148-018-0505-0 (PMC5977551; doi:10.1186/s13148-018-0505-0)
Supplement: Supplementary file 1 — Table S1. Characteristics of antibodies used in immunohistochemical staining. (PDF 499 kb) [file 13148_2018_505_MOESM1_ESM.pdf]

Lp.	Antibody	Manufacturer Catalog No	Clonality	Immunogen	Control	Dilution / incubation
**1.**	**Anti-TET1**	Gene Tex Cat.No.: GTX627420	Mouse Monoclonal	Recombinant protein encompassing a sequence within the center region of human TET1.	small intestine	1:100 / 16h 4°C
**2.**	**Anti-TET2**	Gene Tex Cat.No.: GTX124205	Rabbit Polyclonal	Recombinant protein encompassing a sequence within the N-terminus region of human TET2.	small intestine	1:500 / 16h 4°C
**3.**	**Anti-TET3**	Gene Tex Cat.No.: GTX121453	Rabbit Polyclonal	Recombinant protein encompassing a sequence within the C-terminus region of human TET3.	small intestine	1:500 / 16h 4°C

Table S1. Characteristics of antibodies used in immunohistochemical staining
