# Supplementary material for: Characteristic profiles of DNA epigenetic modifications in colon cancer and its predisposing conditions—benign adenomas and inflammatory bowel disease
Source: Clin Epigenetics. 2018 May 30;10:72. doi: 10.1186/s13148-018-0505-0 (PMC5977551; doi:10.1186/s13148-018-0505-0)
Supplement: Supplementary file 2 — Table S2. Transition patterns specific detector settings and sources of standards for analyzed compounds (relative response ratio = area under the peak of qualifierion/area under the peak of quantifier ion). (PDF 82 kb) [file 13148_2018_505_MOESM2_ESM.pdf]

**compound name**		**relative response ratio**	**ionization mode**	**nominal molecular mass (Da)**	**pseudomolecular ion formulation**	**nominal parent ion (Da)**	**nominal daughter ion (Da)**	**capillary (kV)**	**cone (V)**	**collision (eV)**	**standard source**
5-(hydroxymethyl)-2'-deoxycytidine	quantifier	0.033	ESI+	257	[M+H]+	258	124	1.2	15	10	Berry & Associates, Dexter, MI, USA
	qualifier		ESI+	257	[M+H]+	258	142	1.2	15	10	
[D_3]-5-(hydroxymethyl)-2'-deoxycytidine	quantifier	0.031	ESI+	260	[(M+3)+H]+	261	127	1.2	15	10	Toronto Research Chemicals, Toronto, Canada
	qualifier		ESI+	260	[(M+3)+H]+	261	145	1.2	15	10	
5-formyl-2'-deoxycytidine	quantifier	0.182	ESI-	255	[M-H]-	254	121	3.5	28	18	Berry & Associates, Dexter, MI, USA
	qualifier		ESI-	255	[M-H]-	254	138	3.5	28	18	
[$^{13}C_{10}$, $^{15}N_2$]-5-formyl-2'-deoxycytidine	quantifier	0.178	ESI-	267	[(M+12)-H]-	266	128	3.5	28	18	own synthesis, see M&M section
	qualifier		ESI-	267	[(M+12)-H]-	266	145	3.5	28	18	
5-carboxy-2'-deoxycytidine	quantifier	0.318	ESI-	271	[M-H]-	270	110	3.5	20	20	Berry & Associates, Dexter, MI, USA
	qualifier		ESI-	271	[M-H]-	270	93	3.5	20	20	
[$^{13}C_{10}$, $^{15}N_2$]-5-carboxy-2'-deoxycytidine	quantifier	0.300	ESI-	283	[(M+12)-H]-	282	116	3.5	20	20	own synthesis, see M&M section
	qualifier		ESI-	283	[(M+12)-H]-	282	99	3.5	20	20	
5-(hydroxymethyl)-2'-deoxyuridine	quantifier	1.092	ESI-	258	[M-H]-	257	124	3.5	20	15	Berry & Associates, Dexter, MI, USA
	qualifier		ESI-	258	[M-H]-	257	214	3.5	20	10	
[$^{13}C_{10}$, $^{15}N_2$]-5-(hydroxymethyl)-2'-deoxyuridine	quantifier	1.233	ESI-	270	[(M+12)-H]-	269	131	3.5	20	15	own synthesis, see M&M section
	qualifier		ESI-	270	[(M+12)-H]-	269	224	3.5	20	10	
8-oxo-2'-deoxyguanosine	quantifier	0.280	ESI+	283	[M+H]+	284	168	1.2	20	15	Sigma-Aldrich, St. Louis, MO, USA
	qualifier		ESI+	283	[M+H]+	284	140	1.2	20	30	
[$^{15}N_5$]-8-oxo-2'-deoxyguanosine	quantifier	0.292	ESI+	288	[(M+5)+H]+	289	173	1.2	20	15	Cambridge Isotope Laboratories, Tewksbury, MA, USA
	qualifier		ESI+	288	[(M+5)+H]+	289	145	1.2	20	30	

Table. S2. Transition patterns specific detector settings and sources of standards for analysed compounds (relative response ratio=area under the peak of qualifier ion/ area under the peak of quantifier ion)
