# Supplementary material for: Characteristic profiles of DNA epigenetic modifications in colon cancer and its predisposing conditions—benign adenomas and inflammatory bowel disease
Source: Clin Epigenetics. 2018 May 30;10:72. doi: 10.1186/s13148-018-0505-0 (PMC5977551; doi:10.1186/s13148-018-0505-0)
Supplement: Supplementary file 3 — Table S3. Primers and short hydrolysis probes used for TETs and AID mRNA expression analysis. (PDF 442 kb) [file 13148_2018_505_MOESM3_ESM.pdf]

Gene	Forward primer sequence	Reverse primer sequence	UPL
TET1	5'-TCTGTTGTTGTGCCTCTGGA-3'	5'-GCCTTTAAAACTTTGGGCTTC-3'	#57
TET2	5'-GCCTTTGCTCCTGTTGAGTT-3'	5'-ACAAGGCTGCCCTCTAGTTG-3'	#38
TET3	5'-CACTCCGGAGAAGATCAAGC-3'	5'-GGACAATCCACCCTTCAGAG-3'	#1
AID	5'-GACTTTGGTTATCTTCGCAATAAGA-3'	5'-AGGTCCCAGTCCGAGATGTA-3'	#69

Table S3. Primers and short hydrolysis probes used for *TETs* and *AID* mRNA expression analysis.
